# A real-world study of the optimal cut-off value for vancomycin trough concentration associated with outcomes in children infected with drug-resistant Gram-positive bacteria

**DOI:** 10.3389/fped.2025.1597306

**Published:** 2025-07-21

**Authors:** Yinghui Yan, Manli Wang, Mi Zhou, Jingxing Yang, Zengyan Zhu, Fengjiao Wang

**Affiliations:** ^1^ Department of Pharmacy, Children’s Hospital of Soochow University, Suzhou, Jiangsu, China; ^2^ Department of Neurology, Children’s Hospital of Soochow University, Suzhou, Jiangsu, China; ^3^ National Key Laboratory of Immunity and Inflammation, Suzhou Institute of Systems Medicine, Chinese Academy of Medical Sciences & Peking Union Medical College, Suzhou, Jiangsu, China; ^4^ Key Laboratory of Synthetic Biology Regulatory Element, Institute of Systems Medicine, Chinese Academy of Medical Sciences and Peking Union Medical College, Suzhou, Jiangsu, China

**Keywords:** children, vancomycin, trough concentrations, outcomes, optimal cut-off value

## Abstract

**Background:**

Due to a lack of studies on the relationship between vancomycin trough concentration and clinical outcomes in pediatric patients, there is insufficient evidence to provide a unified standard for vancomycin trough concentration for children.

**Methods:**

We retrospectively analyzed the data of drug-resistant Gram-positive bacteria isolated from human germfree samples of 66 children diagnosed as definite infectious diseases. Vancomycin was intravenously delivered and the trough concentration was monitored regularly. Receiver operator characteristic curve (ROC curve) was used to explore the relationship between vancomycin trough concentration and treatment outcome.

**Results:**

40.9% of the enrolled pediatric patients had poor outcomes. A vancomycin trough concentration above 6.8 mg/L (OR = 0.014, 95% confidence interval 0.001–0.351, *P* = 0.009) was identified as an independent protective factor, while trough concentrations above 10 mg/L appeared to be necessary to support favorable outcomes within 4 days of treatment in children with secondary bloodstream infections and non-bloodstream infections. 4 (6.35%) patients displayed vancomycin-related acute kidney injury (AKI) with an average trough concentration of 10.85 mg/L, and 50% of them simultaneously used nephrotoxic drugs. Moreover, within 7 days of vancomycin administration, there was a significant decrease in serum creatinine and an increase in creatinine clearance rate, and the children with augmented renal clearance exhibited significantly lower vancomycin trough concentrations and higher proportion of poor outcomes.

**Conclusion:**

A vancomycin trough concentration above 6.8 mg/L is sufficient to support favorable outcomes in children who were infected with drug-resistant Gram-positive bacteria. Compared with vancomycin-associated AKI, augmented renal clearance and subsequent poor antibiotic treatment outcome deserve more attention.

## Introduction

Vancomycin is a glycopeptide antibiotic mainly used for treating severe Gram-positive bacterial infection. It is of concern that the therapeutic window for vancomycin is narrow, and therapeutic drug monitoring (TDM) of vancomycin has been proposed to maximize its clinical efficacy and reduce its nephrotoxicity (1). Vancomycin trough concentrations recommended by previous guidelines from the Infectious Diseases Society of America (IDSA) typically ranged from 10 to 20 mg/L for adults (2), and the latest guideline recommend an area under the curve to minimal inhibitory concentration (AUC/MIC) ratio between 400 and 600 (assuming a vancomycin MIC of 1 mg/L) to achieve clinical efficacy while improving patient safety (3). Several cohort studies (4–7) have shown that compared to trough-based administration, AUC-based administration of vancomycin is significantly associated with a reduced risk of nephrotoxicity, while no differences were found in treatment failure and mortality. Due to the potential difficulty in availability of a Bayesian approach and the feasibility of collecting two steady-state samples, trough concentration and AUC measurements are equally recommended by the guidelines of the Chinese Pharmacological Society (1).

Compared to adults, pediatric patients pose additional complexity for managing vancomycin trough concentration range, owing to their continual maturation of glomerular filtration, which is directly related to vancomycin clearance rate (3). For such reasons, it is controversial to take reference of the trough concentration range for adults for the treatment of children. However, there are few studies evaluating the relationship between clinical efficacy and trough concentration of vancomycin in children (8), and the latest guideline recommended trough concentration of 5–15 mg/L for pediatric patients was based on low-quality evidence (1). The lack of reliable trough concentration guidelines brings significant challenges for safe and effective administration of vancomycin in pediatric patients.

This study analyzes the relationship between vancomycin trough concentration and treatment outcome of infection by drug-resistant Gram-positive bacteria in children using real-world data, as well as exploring the influencing factors, so as to determine the optimal cut-off value of vancomycin trough concentration and provide evidence for the optimal range of vancomycin therapeutic drug monitoring for pediatric patients.

## Materials and methods

### Study design

**Figure 1:**
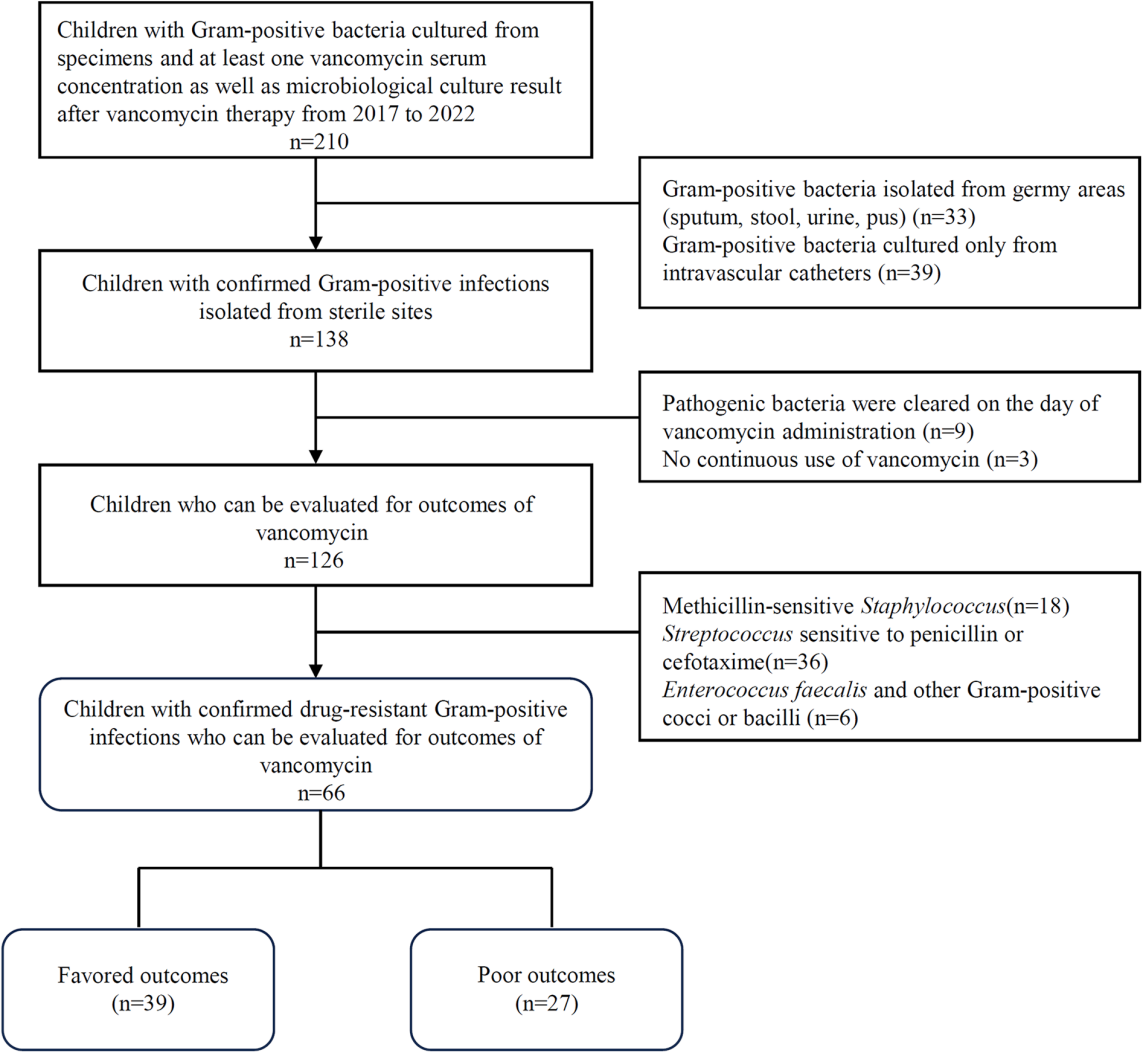
Inclusion and exclusion criteria of participants.

Data were collected from pediatric patients who were hospitalized at the Children's Hospital of Soochow University from 2017 to 2022. Patients were intravenously infused with vancomycin for a confirmed Gram-positive infection and had at least one vancomycin serum concentration that was assayed for TDM. Patients who met all the following criteria (Figure 1) were enrolled: (i) children younger than 18 years old (neonates included); (ii) children with available data on initial trough concentrations obtained after 4 doses of intravenous vancomycin; and (iii) children with Gram-positive bacteria isolated from sterile sites and at least one microbiological culture result from the same site during vancomycin therapy. Additionally, patients were excluded when they met any one of the following criteria: (i) Gram-positive bacteria isolated from germy areas (sputum, stool, urine, pus); (ii) Gram-positive bacteria cultured only from intravascular catheters; (iii) pathogenic bacteria were cleared on the day of vancomycin administration or no continuous use of vancomycin; and (iv) pathogenic bacteria were sensitive to methicillin, penicillin or cephalosporins and vancomycin was not the preferred treatment drug. Covariates were extracted from medical records, including sex, age, weight, body mass index (BMI), vancomycin dosage, treatment duration and trough concentration of vancomycin, other antimicrobials against Gram-positive bacteria used, comorbidities, bacterial strain and drug resistance, and infection type. According to the growth standard for children of China (9, 10), BMI between P15 and P85 is defined as normal range, and BMI below P15 or above P85 is defined as low or high range, respectively. Neonates were defined as babies younger than 4 weeks, whether born at term or prematurely. Premature infant was defined as live birth born before 37 full weeks of gestational age (11).

### Patient and infection profiles

The enrolled cases were divided into those with bloodstream infections (BSI) or non-bloodstream infections, according to whether their blood cultures were positive with signs of systemic infection or not (12). Cases with bloodstream infections were further divided into those with primary bloodstream infections, secondary bloodstream infections or central line-associated bloodstream infections (CLABSI), according to the origin of infection (https://www.cdc.gov/nhsn/pdfs/pscmanual/4psc_clabscurrent.pdf accessed January 2024). In our study, a primary bloodstream infection was defined as a laboratory-confirmed bloodstream infection that is not secondary to an infection at another body site. A secondary bloodstream infection is thought to be seeded from a site-specific infection at another body site, and in our study it mainly included two scenarios: (i) culture results of cerebrospinal fluid, bone marrow and pleural fluid were positive before blood culture and were later matched with that of blood culture; (ii) prior to positive reports of blood culture, there had been confirmed infection of other specific sites (13), and blood specimen was collected for culture during the site-specific infection window period. Central line-associated BSI (CLABSI) was identified when results of both central line [peripherally inserted central venous catheter (PICC) and PORT in our study] and peripheral blood cultures were positive, and the positive result of the central line culture was prior to that of the peripheral blood culture.

### Dosing, sampling and serum assay of vancomycin

Vancomycin was administered as an intravenous infusion over 60 min. The empirical initial dosing regimen was 40–60 mg/kg/day that was divided into 3 or 4 doses, and for older children with a larger body weight, the dosing regimen may be reduced to 2 doses per day. Vancomycin trough concentrations were monitored 30 min before the fifth or sixth doses of intravenous vancomycin to obtain the steady-state trough concentration. Serum vancomycin concentrations were determined by fluorescence polarization immunoassay (FPIA) using an automatic biochemical analyser (Viva-E, Siemens, Berlin, Germany).

### Clinical outcome and adverse effect evaluation

Evaluation criteria for clinical outcomes were determined jointly by pediatricians and clinical pharmacists. Microbial clearance time was determined in combination with the follow-up results of culture. The follow-up time for different sites varied depending on the difficulty of specimen acquisition. Blood culture was followed up for approximately 3–5 days, while cerebrospinal fluid, bone marrow, and puncture fluid culture were followed up for approximately 5–14 days. For BSI, microbial clearance time was calculated based on the clearance of bacteria in blood cultures. Poor outcomes after vancomycin treatment in enrolled children were defined by (i) time from vancomycin administration to microbial clearance >4 days and febrile (temperature >38°C) time ≥4 days and (ii) growth of the same pathogen as in the initial culture within 30 days (relapsed infection). Favored outcomes were defined by (i) time from vancomycin administration to microbial clearance ≤4 days; (ii) time from vancomycin administration to microbial clearance time ≥5 days (due to the difficulty in obtaining specimens) but febrile (temperature >38°C) time ≤3 days; and (iii) no relapsed infection. According to these criteria, the outcome of each enrolled patient was scored as favored or poor, which was then converted into a binary categorical variable, with poor outcomes recorded as “1” and favored outcomes as “0”. The subsequent univariate or multivariate analyses were based on this variable.

Adverse effects (AEs) during vancomycin therapy were recorded in all enrolled children and evaluated for their relationship with vancomycin administration. We used a modified Schwartz formula eGFR=41.3×[height(meter)/Scr(mg/dL)] (14) to estimate the glomerular filtration rates before vancomycin administration (baseline), on the day of the initial TDM sample collection and 2 or 7 days after vancomycin administration for each patient. Occurrence of acute kidney injury (AKI) after vancomycin administration was identified by either an increase in serum creatinine (Scr) of ≥26.5 µmol/L within 48 h or a known or presumed increase in serum creatinine to ≥1.5 times than baseline within the previous 7 days or a urine volume <0.5 mL/kg/h for 6 h (15). Augmented renal clearance (ARC) was defined as a creatinine clearance (CrCl) ≥130 mL/min/1.73 m^2^ (16).

### Statistical analysis

All statistical analyses were performed using IBM SPSS Statistics 26 (International Business Machines Corporation, Armonk, New York, USA), and figures were generated by GraphPad Prism 8 (GraphPad Software, San Diego, California, USA). For quantitative data, normality analysis was performed by the Shapiro–Wilk test, and data were represented by means ± standard deviation, with comparison by *t* test or one-way ANOVA. Quantitative data not conforming to a normal distribution were represented by the median (lower quartile, upper quartile), with comparisons by the Mann‒Whitney *U* test, Kruskal–Wallis test, and Wilcoxon matched-pairs signed rank test. Categorical variables were represented by the number of cases (constituent ratio), and the comparison between groups was completed through the chi-square test. Correlations for all analyses were assessed at a significance level of *α* = 0.05. For the establishment of binary logistic regression models, the primary outcome of interest was whether the outcome of vancomycin treatment was favored or poor, and covariates with significant differences (*P* ≤ 0.1) in the univariate analysis were selected for inclusion in the multivariate analysis. Goodness of fit was tested by the Hosmer Lemeshow test, and the likelihood ratio test was used to screen variables and compare the models.

## Results

### Vancomycin trough concentration and outcome

The baseline characteristics of the 66 enrolled children were shown in Table 1. According to the criteria for poor clinical outcome in this study, children were divided into the poor outcome group and the favored outcome group. 40.90% of the enrolled children had poor outcomes (Table 1). Compared with the favored outcome group, dose of vancomycin administrated in the poor outcome group was similar, but the trough concentration of vancomycin was significantly lower (*P* = 0.001). In addition, in the univariate analysis, outcome of vancomycin treatment was also related to height, whether the patient was a neonate, and the types of infection and pathogenic bacteria.

**Table 1:** The baseline characteristics of children included in this study.

Variates	Total (66)	Favored outcomes (39)	Poor outcomes (27)	
	Median (P25, P75)/*N* (%)			*p*-value
Sex (male)	30 (45.45)	16 (41.03)	14 (51.85)	0.385
Age (years)	2.5 (0.10, 8.18)	1.5 (0.00, 8.00)	3.8 (1.0, 7.95)	0.062
Height (cm)	91.50 (50.50, 124.25)	77.0 (48.00, 119.50)	101.0 (76.00, 131.00)	**0**.**046**
Weight (kg)	12.45 (3.33, 23.90)	9.20 (2.75, 22.50)	15.00 (8.85, 25.10)	0.094
BMI				0.669
Low BMI	33 (50.00)	18 (46.15)	15 (55.56)	
Normal BMI	23 (34.85)	14 (35.90)	9 (33.33)	
High BMI	10 (15.15)	7 (17.95)	3 (11.11)	
Neonate	18 (27.27)	15 (38.46)	3 (11.11)	**0**.**014**
Weight (kg)	2.75 (0.97, 3.327)			
Premature infant	10 (15.15)			
Weight (kg)	0.98 (0.865, 1.285)			
Vancomycin dose (mg/kg/day)	40.00 (37.74, 45.23)	40.00 (37.27, 44.58)	40.91 (38.84, 45.29)	0.460
Vancomycin trough concentrations (mg/L)	6.20 (3.50, 11.65)	8.90 (4.30, 12.70)	4.10 (2.65, 6.45)	**0**.**001**
Combination regimens	7 (10.61)	3 (7.69)	4 (14.81)	0.605
Infection type				**<0**.**001**
Primary bloodstream infection	38 (57.58)	28 (71.79)	10 (37.04)	
Secondary bloodstream infection	14 (21.21)	4 (10.26)	10 (37.04)	
CLABSI	9 (13.64)	7 (17.95)	2 (7.41)	
Non-bloodstream infection	5 (7.58)	0 (0)	5 (18.52)	
Comorbidities				0.108
Hematologic malignancy	25 (37.88)	15 (38.5)	10 (37.0)	
Abnormal liver and kidney functions	5 (7.58)	3 (7.7)	2 (7.4)	
Premature infant	10 (15.15)	9 (23.08)	1 (3.70)	
Other diseases (non-risk factors)	26 (39.39)	12 (30.77)	14 (51.85)	
Pathogenic bacteria				**0**.**02**
Methicillin-resistant *Staphylococcus*	44 (66.67)	31 (79.49)	13 (48.15)	
*Streptococcus* (Penicillin R, Cefotaxime R)	17 (25.76)	7 (17.95)	10 (37.04)	
*Enterococcus faecium*	5 (7.58)	1 (2.56)	4 (14.81)	

Bold values indicate statistically significant differences (*P* < 0.05).

BMI, body mass index; CLABSI, central line-associated bloodstream infection.

**Figure 2:**
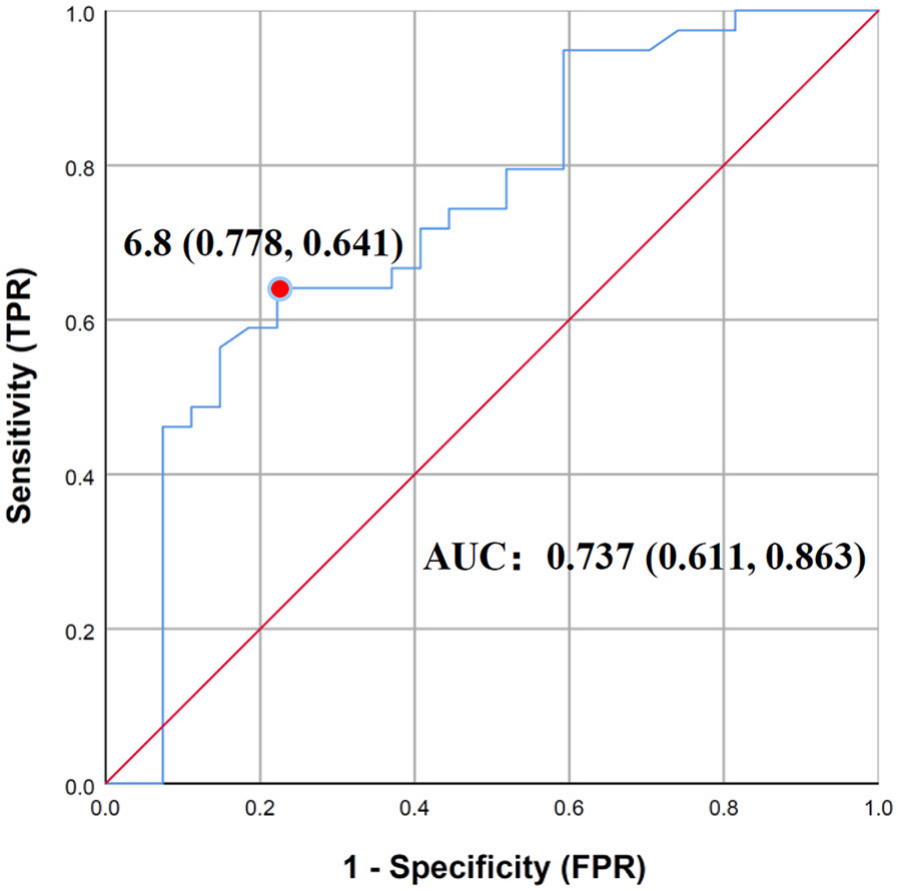
Receiver operating characteristic curve of vancomycin trough concentration and outcomes (*n* = 66).

To further determine the risk factors related to poor clinical outcomes, the covariates with a *P* value ≤ 0.10 in the univariate analysis were included in the multiple logistic regression analysis. Prior collinearity diagnosis showed that age was moderately collinear with height and weight, so only age was included in the model. In addition, owing to no linear relationship with logit conversion value of the dependent variable, vancomycin trough concentration had to be converted to a categorical variable. It is worth noting that we did not choose 5 mg/L or 10 mg/L that were suggested on low-quality evidence or for adults as the cut-off value, but instead focused on finding a more realistic cut-off in the enrolled children. Therefore, based on the evaluation method of diagnostic tests, we took outcome as the gold standard and established the receiver operating characteristic curve (ROC curve) in order to find best cut-off value of vancomycin trough concentration, which could predict antibacterial outcomes, as shown in Figure 2. The area under ROC curve was 0.737 (*P* = 0.001), and when the Youden index was maximum, the cut-off value of vancomycin trough concentration was 6.8 mg/L, with sensitivity and specificity being 0.641 and 0.778. Based on the results above, vancomycin trough concentration was converted into categorical variable with 6.8 mg/L as cut-off value and put into multiple regression model with age, neonates or not, types of infection and pathogenic bacteria, and the results with Enter Method were optimal and shown in Table 2. Evidently, vancomycin trough concentration above 6.8 mg/L was the only variate significantly associated with favored outcomes, with a hazard ratio of 0.014.

**Table 2:** Multivariate analysis results of vancomycin clinical outcomes.

Variates	Wald	SE	OR (95%CI)	*p*-value
Age	2.142	0.156	1.257 (0.925–1.708)	0.143
Neonates^a^	0.00	6,885.11	0.00 (0.00−)	0.998
Vancomycin trough concentrations (≥6.8 mg/L)^b^	6.732	1.649	0.014 (0.001–0.351)	**0** **.** **009**
Infection type^c^	0.013			1
Secondary bloodstream infection	0.00	6,885.11	1.70E + 09 (0.00−)	0.998
CLABSI	0.013	1.059	1.130 (0.142–9.00)	0.908
Non-bloodstream infection	0.00	14,869.8	7.38E + 18 (0.00−)	0.998
Pathogenic bacteria^d^	3.434			0.18
*Streptococcus* (Penicillin R, Cefotaxime R)	0.038	0.928	1.198 (0.194–7.391)	0.846
*Enterococcus faecium*	3.396	1.914	34.063 (0.799–1,451.78)	0.065

Bold values indicate statistically significant differences (*P* < 0.05).

CLABSI, central line-associated bloodstream infection.

^a^ Control: non-neonates.

^b^ Control: vancomycin trough concentrations (<6.8 mg/L).

^c^ Control: primary bloodstream infection.

^d^ Control: methicillin-resistant *Staphylococcus.*

**Figure 3:**
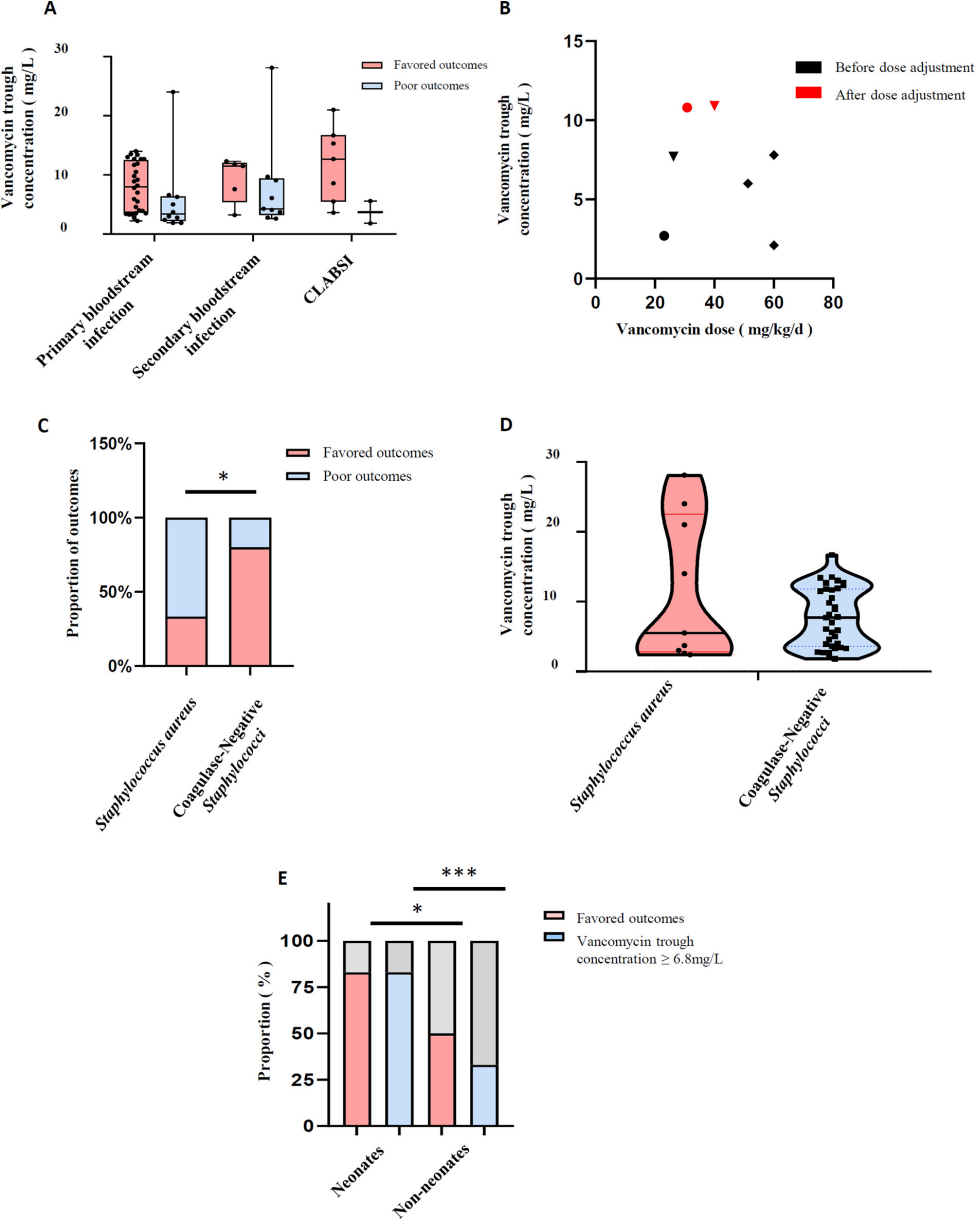
Vancomycin trough concentration and outcomes. **(A)** Vancomycin trough concentration and outcomes in different infection types; **(B)** trough concentration and dose of vancomycin in children with non-bloodstream infection; **(C)** comparison of outcomes in patients with methicillin-resistant *Staphylococcus*; **(D)** comparison of vancomycin trough concentrations in patients with methicillin-resistant *Staphylococcus*; **(E)** outcomes and vancomycin trough concentrations in neonates and non-neonates. *P < 0.05, **P < 0.01, ***P < 0.001.

Since secondary bloodstream infection and non-bloodstream infection were significantly correlated with poor outcomes in the univariate analysis, and the outcome of neonates was significantly better, which resulted in height as a significant covariate, we paid attention to the relationship between vancomycin trough concentration and outcome in different types of infection and pathogenic bacteria, as well as neonates or not, which were shown in Figure 3. The trough concentration of vancomycin was relatively higher in patients with secondary bloodstream infection and CLABSI with favored outcomes compared with primary bloodstream infection, although there was no statistical difference (11.65 mg/L and 12.7 mg/L vs. 8.0 mg/L, *P* = 0.18 in Figure 3A). In the non-bloodstream infection group shown in Figure 3B, all five children had poor outcomes, with a median vancomycin dose of 51 mg/kg/day and a median trough concentration of 6 mg/L, and the maximum value was 7.8 mg/L. Among them, the trough concentration of two patients with vancomycin doses lower than 40 mg/kg/day reached 10.8 mg/L and 10.9 mg/L respectively after dose adjustment, and their body temperature returned to normal the next day after dose adjustment. For the three distinct types of drug-resistant pathogens, while the outcomes appeared to differ in the univariate analysis, there were no significant differences in vancomycin trough concentrations (7.0 mg/L, 5.75 mg/L and 7.6 mg/L for Methicillin-resistant *Staphylococcus*, *Streptococcus* and *Enterococcus faecium*, respectively, *P* = 0.442). Notably, for Methicillin-resistant *Staphylococcus aureus* and coagulase-negative *Staphylococci*, the latter exhibited a more favorable outcome (80% vs. 33.33%, *P* = 0.02 in Figure 3C); however, the vancomycin trough concentrations remained no statistical difference (7.7 mg/L in coagulase-negative *Staphylococci* vs. 5.5 mg/L in *Staphylococcus aureus*, *P* = 0.73 in Figure 3D). Interestingly, in Figure 3E, better outcomes in neonates (83.3% vs.50%, *P* = 0.014) were associated with a higher proportion of children having vancomycin trough concentrations above 6.8 mg/L (83.3% vs.33.3%, *P* < 0.001). The analysis in Table 3 showed that neonates achieved significantly higher trough concentrations at similar vancomycin doses. Although the baseline serum creatinine levels of neonates were similar with non-neonates, the estimated glomerular filtration rate was significantly lower (*P* < 0.001).

**Table 3:** Comparison of vancomycin treatment and renal function between neonates and non-neonates.

Variates	Neonates	Non-neonates	*p*-value
Vancomycin dose (mg/kg/day)	40.69 (28.94, 46.21)	40.0 (38.52, 45.40)	0.840
Vancomycin trough concentrations (mg/L)	9.50 (7.68, 12.0)	4.45 (2.85, 9.43)	**0** **.** **004**
Serum creatinine (μmol/L)	26.15 (16.63, 43.45)	26.85 (19.33, 39.45)	0.851
eGFR (mL/min/1.73 m^2^)	56.57 (33.93, 87.19)	141.02 (116.43, 171.49)	**<0**.**001**

Bold values indicate statistically significant differences (*P* < 0.05).

### Vancomycin trough concentration and renal function

**Figure 4:**
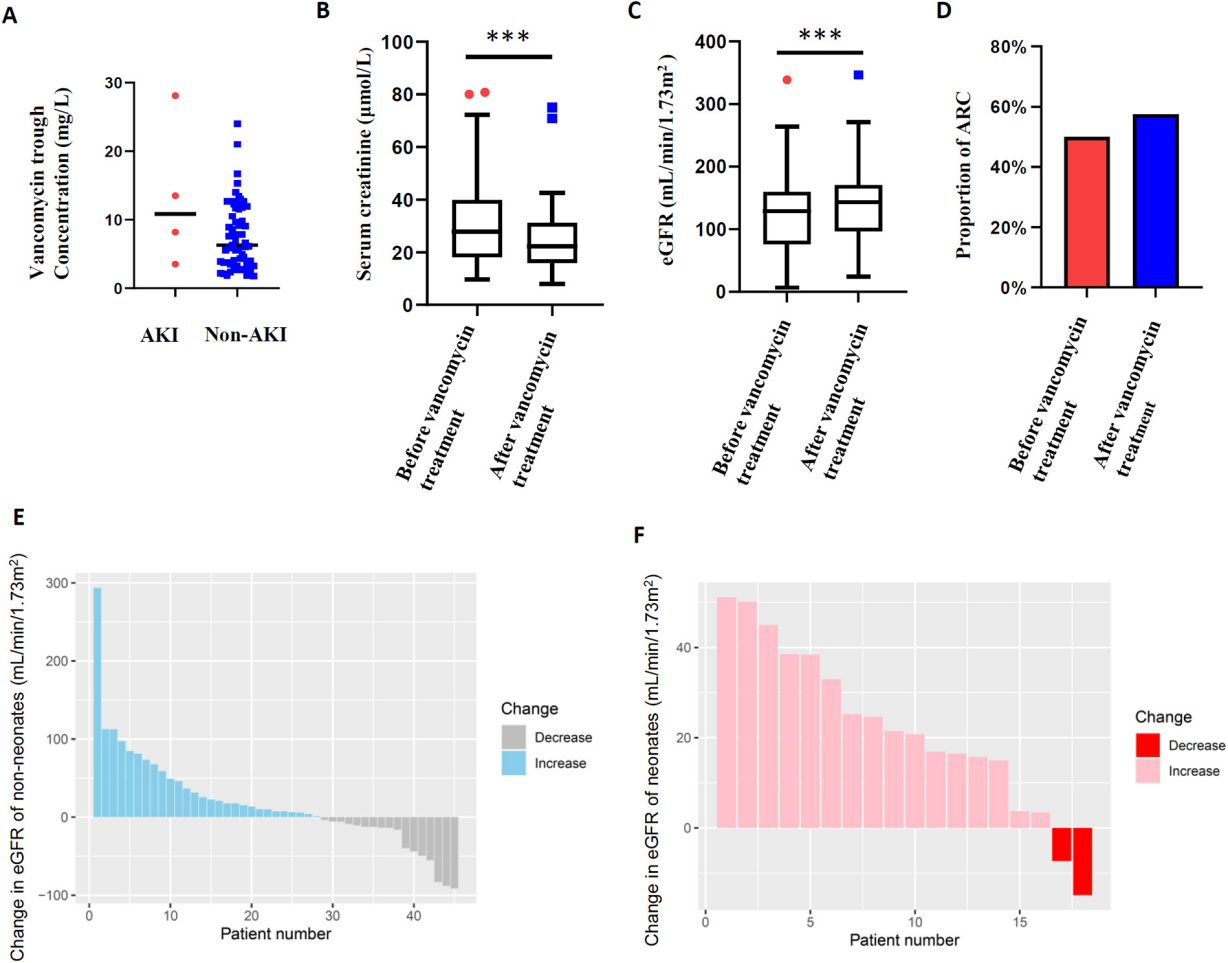
Vancomycin trough concentrations and renal function. **(A)** AKI and vancomycin trough concentration; **(B)** serum creatinine levels in children within 7 days of vancomycin administration; **(C)** estimated glomerular filtration rate in children within 7 days of vancomycin administration; **(D)** proportion of ARC in children within 7 days of vancomycin administration; **(E)** change in eGFR within 7 days of vancomycin administration in non-neonates; **(F)** change in eGFR within 7 days of vancomycin administration in neonates. *P < 0.05, **P < 0.01, ***P < 0.001.

**Figure 5:**
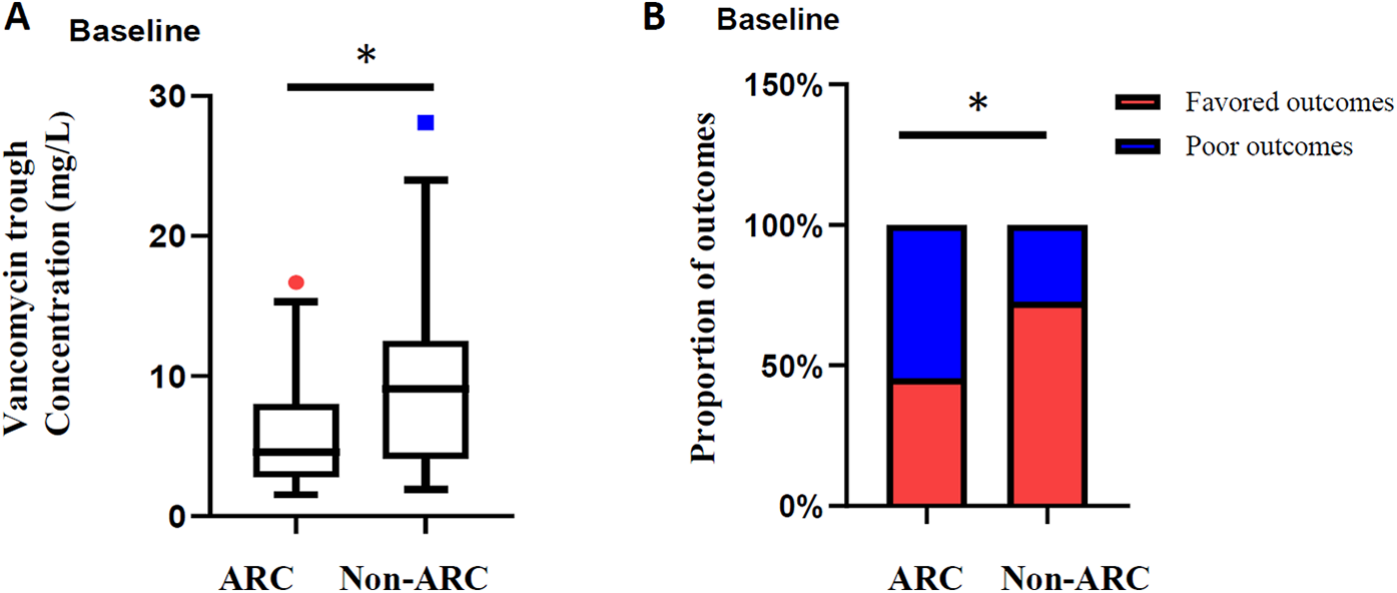
**(A)** Initial vancomycin trough concentrations in children with ARC or not at baseline; **(B)** outcome comparison in children with ARC or not at baseline. *P < 0.05, **P < 0.01, ***P < 0.001.

To assess the renal safety of vancomycin, renal function before and during vancomycin treatment of 63 pediatric patients was analyzed, excluding 3 patients missing data on renal function after vancomycin treatment. Undoubtedly, acute kidney injury is a major concern for vancomycin administration. Ultimately, 4 (6.35%) patients showed vancomycin-related AKI among all 63 patients. The median vancomycin trough concentration of 4 patients was 10.85 mg/L compared with 6.05 mg/L in the non-AKI group (*P* = 0.175, Figure 4A). In addition, 2 of the 4 patients were treated respectively with tacrolimus and furosemide in combination. Adverse effects of vancomycin were recorded in 2 (3.03%) of the patients (rash 1, ototoxicity 1). Moreover, our previous studies have shown that augmented renal clearance is the primary cause of vancomycin suboptimal trough concentration in children, and in this study the serum creatinine levels in 63 patients were significantly lower than baseline within 7 days of vancomycin administration (22.2 µmol/L vs. 27.8 µmol/L, *P* = 0.001) (Figure 4B), with the eGFR level increasing from 128.8 mL/min/1.73 m^2^ to 143.2 mL/min/1.73 m^2^ (*P* = 0.001, Figure 4C). Based on this, we analyzed the proportion of children with ARC, and the ratio showed an increase within 7 days of vancomycin administration (49.2% vs. 60.3%, *P* = 0.210, Figure 4D). Furthermore, the serum creatinine levels of neonates (including preterm infants) are significantly higher than that of other age groups (17), and the glomerular filtration rates increase significantly with age. In order to avoid the interference of renal function fluctuations in neonates, we showed the change of eGFR within 7 days of vancomycin treatment in the enrolled children by stratification according to whether neonates or not. As can be seen in Figure 4E, eGFR of 62.22% non-neonates (*n* = 45) increased with a median increase of 24.09 mL/min/1.73 m^2^; while eGFR increased in 88.89% of neonates (*n* = 18), with a median increase of 23.04 mL/min/1.73 m^2^ (Figure 4F). Above all, the difference in renal clearance was also reflected in the vancomycin concentration and treatment outcome. The initial vancomycin trough concentration in children with ARC at baseline was significantly lower than that in the non-ARC group (4.6 mg/L vs. 8.9 mg/L, *P* = 0.017, Figure 5A), and a significantly higher proportion of children with ARC had poor outcomes (54.5% vs. 27.3%, *P* = 0.024, Figure 5B).

## Discussion

Lack of high-quality original studies is one of the main reasons why there has been no consensus on the optimal trough concentration of vancomycin in pediatric patients. In this study, we retrospectively analyzed the data of 66 children with drug-resistant Gram-positive bacterial infection, and found that 6.8 mg/L was the best cut-off value of vancomycin trough concentration for favored anti-infective outcomes. Notably, vancomycin trough concentrations greater than 6.8 mg/L was the only protective factor for poor outcomes, while for secondary bloodstream infection and non-bloodstream infection, vancomycin trough concentrations higher than 10 mg/L may be required to ensure fever reduction or pathogen cleared within 4 days. Neonates had lower renal clearance rates and higher trough concentrations of vancomycin at the dosage indicated on the label, thus having a better outcome. Acute kidney injury related to vancomycin often occurs when multiple drugs that can cause renal damage were used simultaneously, while augmented renal clearance was an important factor that may trigger suboptimal trough concentration of vancomycin and even poor outcomes in pediatric patients.

While current guidelines increasingly favor AUC-guided monitoring for vancomycin in adults (3), evidence remains insufficient for specific recommendations for pediatric patients. Although pharmacokinetic modeling studies are emerging to address this gap, challenges including high heterogeneity among pediatric patients, limited feasibility of multiple blood sampling, and immature renal function continue to restrict the clinical application in children. Consequently, monitoring of trough concentration is still widely used to guide dose adjustment of vancomycin. For pediatric patients, evidence-based guideline from the Chinese Pharmacological Society recommended a stable vancomycin trough concentration of 5–15 mg/L based on low-quality evidence. One systematic review (18) reported that children with vancomycin trough concentrations >10 mg/mL had a higher clinical efficacy rate and microbial clearance but higher incidences of nephrotoxicity. Whereas, the results of Meta-analysis (18, 19) was restricted to grouping of original studies, and only data below or between cut-off values of 10 mg/L and 15 mg/L could be combined. Given that it is often difficult to achieve a trough concentration of 10 mg/L at the recommended dose of vancomycin in children (8, 20), we grouped children according to outcomes instead of vancomycin trough concentration in this real-world study. Finally, the cut-off value of 6.8 mg/L we found for predicting favorable anti-infective outcomes was ideal with the AUC of ROC being 0.737. Moreover, the high specificity of this cut-off value guarantees the clinical application and the sensitivity of 0.641 was due to the fact that some children with favored outcomes had vancomycin trough concentration lower than 6.8 m/L. Interestingly, the consensus guideline stated that in pediatric patients, a target AUC/MIC ratio of 400 was more readily achievable than it is in adults and correlates to trough concentrations of 7–10 mg/L (3), which was consistent with our research.

Our data showed that vancomycin trough concentrations above 10 mg/L were required for secondary bloodstream infection and non-bloodstream infections to obtain favored outcomes as defined in our study. Considering hydrophilicity and apparent volume of distribution, we can infer that vancomycin is more distributed in plasma, which is more suitable for controlling primary bloodstream infections. In contrast, in secondary bloodstream infections, the clearance of pathogen from the primary focus and surgical procedures can affect the patients’ condition, while the delayed removal of the central catheter affects the patients' outcome in CLABSI. Meanwhile, non-bloodstream infections require the distribution of vancomycin into tissues to achieve better outcomes. Although the latest guidelines (1, 3) did not give a recommended range of vancomycin trough levels for different types of infection and only emphasized that the trough levels in adult patients with severe methicillin-resistant *Staphylococcus aureus* (MRSA) infection should be maintained at 10–20 mg/L (1), we still found a link between vancomycin trough concentration and outcomes in different types of infection. For primary bloodstream infections, the trough concentration of vancomycin should be maintained at 6.8 mg/L or above. For secondary and non-bloodstream infections (in this study, central nervous system infections were mainly considered), trough concentrations above 10 mg/L were necessary to ensure fever reduction or pathogen clearance within 4 days. Moreover, when treating CLABSI without removing the central venous catheter, a higher trough concentration (>10 mg/L) should be considered. Regarding different pathogens, our data are insufficient to draw meaningful conclusions. However, our data on vancomycin trough concentrations suggest that it is necessary to study the relationship between outcomes of different pathogens and vancomycin exposure.

Notably, neonates showed lower glomerular filtration rates in our study, resulting in higher vancomycin trough concentrations at the same dose, and thus better outcomes. The guideline (1) of Chinese Pharmacological Society and vancomycin instructions of FDA recommend administering a 15 mg/kg loading dose to neonates, followed by a maintenance dose of 10 mg/kg administered every 12 h or 8 h (up to 7 days of age). This is lower than the recommended dosage in domestic instructions (10–15 mg/kg, every 12 h or 8 h), which has led to generally higher trough concentrations in neonates. Although no adverse reactions were reported in neonates in our study and the outcome was better, the continuously maturing renal function of neonates requires us to strictly monitor vancomycin dosage and trough concentration according to neonatal age in days.

The prevalence of vancomycin-associated AKI reported varied from 5% to 43% (21). Results of meta-analysis (22) showed that the strength of evidence that vancomycin was associated with a higher risk of kidney injury was judged to be moderate, with the relative risk being 2.45 (95% confidence interval, 1.69–3.55) and an attributable risk of 59%. Most AKI attacks occur between 4 and 17 days after the start of treatment. In our study, 6.35% of pediatric patients developed AKI within 7 days of vancomycin treatment, with the median vancomycin dose being 40 mg/kg/day and trough concentration being 10.85 mg/L. Increased trough concentrations of vancomycin are associated with a higher risk of AKI, especially when maintained above 15–20 mg/L (21). For children with AKI in our study, vancomycin was dosed at an appropriate level, and average trough concentration was less than 15 mg/L, but 50% of them were treated with nephrotoxic drugs simultaneously. Concurrent use of aminoglycosides or diuretics has been shown to increase the risk of nephrotoxicity (3). Therefore, for children, vancomycin nephrotoxicity requires intensive monitoring when the trough concentration is greater than 10 mg/L and while nephrotoxic drugs are used.

In addition to renal impairment, we also found unexpected results that there was a decrease in serum creatinine and an increase in creatinine clearance rate in the enrolled children. We calculated and analyzed renal function according to the standards of ARC, and the results showed that the proportion of ARC has increased after vancomycin administration. We excluded an increase in eGFR due to increase of neonatal age in days, and 62.22% of non-neonates experienced an increase in eGFR levels. Our previous study has shown that pediatric patients with haematologic diseases had a high prevalence of ARC (20), and ARC is associated with many risk factors, including trauma, surgery, sepsis, burn, subarachnoid haemorrhage, and haematological malignancy (23). Studies in critically ill children have shown that male gender and antibiotic treatment are independently associated with the occurrence of ARC (24). Claus et al. focused on the dynamics of renal function within 15 days of antimicrobial therapy in critically ill adult patients and showed that the ratio of ARC fluctuated between 25% and 45%. Patients who permanently expressed ARC during antimicrobial therapy had a higher rate of treatment failure than those who briefly expressed ARC (1 day) (33.3% vs. 17.4%) (25). Most of the enrolled children in our study experienced sepsis, with the minority suffering from hematologic malignancies, and the ratio of ARC reached 50% before vancomycin treatment. The trough concentration of vancomycin in children with ARC was significantly lower at 4.6 mg/L than that of the non-ARC group, and the proportion of poor outcomes was significantly higher than that of the non-ARC group with a *P* value of 0.024. Notably, the proportion of ARC increased within 7 days of vancomycin treatment, but it is difficult to assess the relationship between occurrence of ARC and vancomycin. Most important of all, the results suggest that in children receiving vancomycin treatment, antimicrobial underexposure induced by ARC and subsequent poor outcomes may be a more serious concern than the occurrence of AKI.

## Conclusion

In this study, vancomycin trough concentration greater than 6.8 mg/L was found to be associated with favorable outcomes in children with drug-resistant Gram-positive bacterial infections. Compared to primary bloodstream infections, a higher trough concentration of vancomycin greater than 10 mg/L was required in secondary and non-bloodstream infections to obtain favored outcomes within 4 days. The incidence of vancomycin-related AKI was low and often occurred when trough concentrations were greater than 10 mg/L and nephrotoxic drugs were used simultaneously. By contrast, augmented renal clearance and subsequent poor outcomes that occur before or during antibiotic therapy in children should be the more serious concern.

## Limitations

There are still some limitations to our study. First, the study was retrospective and medical intervention had occurred, which would lead to bias. Second, some of the enrolled patients suffered from hematologic malignancy, which resulted in meaningless white blood cell counts as a reference for anti-infective outcomes. In addition, the need for patients to use vancomycin can't be guaranteed.

## Data Availability

The original contributions presented in the study are included in the article/Supplementary Material, further inquiries can be directed to the corresponding authors.
